# Heterologously expressed SacP23, a novel bacteriocin from *Paenibacillus polymyxa* #23, is active against methicillin resistant *Staphylococcus aureus*

**DOI:** 10.1098/rsos.231119

**Published:** 2023-12-20

**Authors:** Châu Minh Khánh, Dong Van Quyen, Thi Thu Hao Van, Robert J. Moore

**Affiliations:** ^1^ School of Science, RMIT University, Bundoora, Victoria 3083, Australia; ^2^ NhaTrang Institute of Technology Research and Application, Vietnam Academy of Science and Technology, 02 Hung Vuong, Loc Tho, Nha Trang, Khanh Hoa, Vietnam; ^3^ Institute of Biotechnology, Vietnam Academy of Science and Technology, 18 Hoang Quoc Viet, Cau Giay, Ha Noi, Vietnam; ^4^ University of Science and Technology of Hanoi, Vietnam Academy of Science and Technology, 18 Hoang Quoc Viet, Cau Giay, Ha Noi, Vietnam

**Keywords:** bacteriocin, sactipeptide, antimicrobial, *Paenibacillus polymyxa*, heterologous expression

## Abstract

Antimicrobial peptides have the potential to be used in a range of applications, including as an alternative to conventional antibiotics for the treatment of bacterial infections of humans and animals. Therefore, there is interest in identifying novel bacteriocins which have desirable physico-chemical properties or antimicrobial activities. *Paenibacillus polymyxa* #23, isolated from a marine sponge, has wide spectrum antimicrobial activity against Gram-negative and Gram-positive bacteria. To explore the basis of this antimicrobial activity, the complete genome sequence of the strain was examined. Multiple genes predicted to encode antimicrobial peptides were identified. One gene was predicted to encode a novel sactipeptide bacteriocin, named SacP23. To confirm that SacP23 does have antimicrobial activity and to explore the antimicrobial spectrum of the peptide it was heterologously expressed in *Bacillus subtilis*. A cluster of eight genes, encoding a full complement of accessory genes as well as the structural gene expressed from the native promoter, was cloned into *B. subtilis* BS54A. The recombinant strain displayed antimicrobial activity against several Gram-positive bacteria, including multi-drug resistant *Staphylococcus aureus*. Heterologous expression of a whole gene cluster offers a powerful way to interrogate and resolve the various antimicrobial activities expressed by native strains that encode multiple compounds of interest.

## 1. Introduction

Bacteriocins are ribosomally synthesized antimicrobial peptides expressed by a wide range of bacteria [1,2]. Bacteriocins have been used in various applications for food preservation and therapeutic applications in animals and humans and are being actively researched [3–6]. Interest in the potential therapeutic applications of bacteriocins has increased as the threat posed by bacterial pathogens that are resistant to conventional antibiotics has grown. Bacteriocins are of interest because some have been shown to have strong activity against clinically important antibiotic resistant pathogens, such as methicillin resistant *Staphylococcus aureus* (MRSA) and vancomycin resistant *Enterococcus faecalis* (VRE) [7]. Bacteriocins generally exhibit antimicrobial activity against a narrower taxonomic spectrum of bacteria compared to conventional antibiotics, while susceptible bacteria are less prone to develop resistance to bacteriocins than to conventional antibiotics [8]. Bacteriocins may provide useful therapeutic alternatives to conventional antibiotics [9], including in applications such as directed modulation of the gut microbiota [10].

It is known that the spore-forming bacteria, such as *Bacillus*, *Paenibacillus*, and *Brevibacillus* species, are producers of a wide variety of antimicrobial molecules, among which are bacteriocins [11,12]. The commonly used classification scheme for *Bacillus*-derived bacteriocins comprises three classes, distinguished based on structure, mode of action, thermal stability, and molecular size. Class I bacteriocins are small (less than 10 kDa) and heat stable and are modified compounds; class II bacteriocins are small (less than 10 kDa) heat stable peptides; and class III bacteriocins are larger (greater than 10 kDa) and heat sensitive proteins. The class I bacteriocins are further divided into subclasses, including lantibiotics, head-to-tail peptides, linear azole containing peptides, lassopeptides, thiopeptides, sactipeptides and glycocins [13,14].

The genetic machinery to produce bacteriocins involves various genes that are usually located together in a biosynthetic gene cluster [15]. The gene composition of bacteriocin biosynthetic gene clusters is diverse, but generally comprises: structural gene(s) encoding precursor bacteriocin(s); gene(s) encoding modification enzyme(s), which are responsible for modify the precursor (in the case of class I bacteriocins); gene(s) encoding transport protein(s) that are necessary for bacteriocin secretion; gene(s) encoding immunity protein(s) to protect the host from their otherwise lethal effect; and regulatory genes to control transcription of the bacteriocin gene cluster. The precursor of a bacteriocin comprises an N-terminal leader sequence and a C-terminal core sequence. Bacteriocin maturation usually involves the cleavage of the leader sequence followed by modifications and secretion of the core sequence.

Different methods have been developed to obtain pure bacteriocins for characterization. Conventional peptide purification has been widely used to obtain bacteriocins from wild-type producer strains. However, some bacteria carry bacteriocin gene clusters that remain unexpressed under standard *in vitro* growth conditions. In these cases, heterologous expression systems have been used to express bacteriocin biosynthetic gene clusters, either in heterologous strains in which the native promoters are active or under the control of strong promoters to avoid the regulatory processes that supress expression in the native strains [16]. Examples of heterologous expression of bacteriocins include the production of pediocin PA-1, BacR1, divercin V41 and piscicolin 126 [17], mersacidin [18], plantaricin ZJ5 [19] and nisin [20], mostly using *Escherichia coli* as the host for expression. The procedures frequently included cloning and expression of a single structural gene encoding precursor bacteriocin that was engineered with an artificial tag sequence to facilitate purification, and cleavage sequences so that the bacteriocin precursor could be matured by *in vitro* digestion with commercial enzymes, rather than maturation by the natural bacteriocin synthesis pathway. To investigate the natural maturation, structure, and properties of bacteriocins, it is often desirable to clone the whole biosynthetic gene cluster carrying the structural gene and all the accessory genes. However, because of the size of the gene clusters and the nature of some of the gene products, this can be a difficult task in widely used host systems such as *E. coli* [21].

Despite the extensive efforts over the last several decades devoted to the identification and characterisation of bacteriocins, novel bacteriocins continue to be found and there still appears to be further opportunities to identify novel compounds. It may be worthwhile to explore bacteria isolated from relatively under-investigated environments as potential sources of new compounds. Recently a collection of *Bacillus* and *Paenibacillus* isolates were recovered from the Vietnam Sea, characterized, and identified as expressing antimicrobial activity against a range of Gram-negative and Gram-positive bacteria [22]. One of the strains, *Paenibacillus polymyxa* #23, had particularly potent antimicrobial activity against a wide range of indicator strains. The extent and potency of antimicrobial activity suggested that the strain might express antimicrobial compounds that have not been identified in other bacteria. The purpose of the current study was to investigate the genetic basis of the antimicrobial activity of *P. polymyxa* #23, determine the coding potential for bacteriocins, and express a novel sactipeptide bacteriocin, SacP23, in a heterologous *Bacillus subtilis* system. The distinguishing feature of sactipeptides is that they contain one or more intramolecular thioether linkages between cysteine residues and the a-carbon of other amino acids [23].

## 2. Material and methods

### 2.1. Bacterial strains and culture conditions

Bacterial strains and plasmids used in this study are listed in table 1. *P. polymyxa* #23 and *B. subtilis* BS54A were cultured in tryptone soy agar (TSA) (Oxoid) or tryptone soy broth (TSB) at 30°C. The *E. coli* strains were propagated aerobically in Luria-Bertani (LB) broth (Oxoid) at 37°C. During cloning and transformation, chloramphenicol was used as a selective antibiotic at a final concentration of 5 µg ml^−1^ for *B. subtilis* and 30 µg ml^−1^ for *E. coli* strains, kanamycin was used at a concentration of 50 µg ml^−1^.

**Table 1.** List of bacterial strains and plasmids.

strains or plasmids	relevant properties/strain name	origins
**bacteria**		
*E. coli* DH5*α*	host for copy control pD202L vectors	RMIT University
*E. coli* Top10	host for copy control pD202L/ insert vectors	RMIT University
*E. coli* JIR702	host for induction multimeric DNA	Monash University
*P. polymyxa* #23	wild-type sactipeptide producing strain	this study
*B. subtilis* BS34A	host for heterologous expression of sactipeptide	Monash University
MRSA 344/2-24	indicator strain for antimicrobial test, *Cm*5R	RMIT University
**plasmids**		
pDLL202	*E. coli* and *B. subtilis* shuttle cloning vector; pCC1BAC origin and pAMbeta1 origin, *Em*R, *Cm*R, *Amp*C (10 153 bp)	Monash University
pDLL202Δ-sacP23	fusion plasmid harbouring ‘whole sactipeptide gene cluster’ (15 497 bp)	this study

### 2.2. Purification and sequencing of *Paenibacillus polymyxa* #23 genomic DNA

*Paenibacillus polymyxa* #23 [22] grown in TSB (Oxoid) was harvested by centrifugation and genomic DNA was extracted using a guanidine thiocyanate-based method [24]. To ensure the quality of the genomic DNA, the NanoDrop spectrophotometer (Thermo Fisher Scientific) was used to measure the DNA concentration and purity, while agarose gel electrophoresis was employed to assess the overall integrity of the DNA. A genomic library was prepared using a Nextera XT DNA Library Preparation Kit (Illumina) following the manufacturer's instructions. The library was sequenced using an Illumina MiSeq instrument with 2 × 300 bp paired-end reads.

### 2.3. Genome assembly and annotation

The paired-end sequence reads were *de novo* assembled using the A5-miseq pipeline with default parameters for bacterial species [25]. The draft genome sequence was annotated using the RAST annotation pipeline for rapid annotation of prokaryotic genomes [26]. Putative genes associated with antimicrobial metabolite biosynthesis were identified using Antibiotics and Secondary Metabolites Analysis Shell (AntiSMASH v5.0) to analyse both non-ribosomally synthesized peptides [21] and ribosomally synthesized peptides [27]. Additionally, BAGEL 4 [28] and the BACTIBASE bacteriocin database [29] were employed to assess ribosomally synthesized and post-translationally modified peptides (RiPPs, or bacteriocins). Identified biosynthetic gene clusters (BGCs) were compared to the ‘Minimum Information about a Biosynthetic Gene Cluster’ (MIBiG) database [30].

### 2.4. Generation of the expression plasmid pDLL202Δ-sacP23

The complete sactipeptide gene cluster was amplified from *P. polymyxa* #23 genomic DNA using the pair of primers G23sac_F (5′-CAGCGAGATCTGATCACGCGCAGTTTACCAGTAGCTGCTC-3′) and G23sac_R primer pair, (5′-GCTAGCGACGTCTAGACTAGCGGGAATTAAGTAGGGGTATAGG-3′). The primers were designed to generate a polymerase chain reaction (PCR) product containing the whole sactipeptide gene cluster flanked by end regions of 20 nucleotides, which overlapped with the terminal regions on the plasmid vector backbone. The 50 µl PCR reaction included 2.5 µl of each 10 mM forward and reverse primer (G23sac_F/G23sac_R), 1.5 µl dimethyl sulfoxide, 25 µl Phusion High-Fidelity PCR Master Mix (M0531S, New England Biolabs), 2 µl genomic DNA (100 ng) and 16.5 µl water. The PCR cycling conditions were 98°C for 30 s, 35 cycles of; 98°C for 10 s, 56°C for 30 s, 72°C for 4.5 min, and a final extension at 72°C for 10 min. The PCR product was purified using the Monarch PCR & DNA Cleanup Kit (T1030S, New England Biolabs).

The expression plasmid was generated by fusing the vector plasmid backbone, pDLL202, and the purified PCR product of the sactipeptide gene cluster using the Gibson assembly method. Briefly, a 20 µl reaction was prepared including 10 µl of NEBuilder HiFi DNA Assembly Master Mix (E2621S, New England Biolabs), approximately 0.15 pmol of insert and plasmid backbone (ratio of 1: 3) and water. The Gibson reaction was incubated at 50°C for 60 min and immediately used for transformation. A truncated version of pDLL202 was used; it was prepared by digesting pDLL202 with the restriction enzymes *Mlu*I and *Spe*I (New England Biolabs) and the digested vector backbone DNA was excised from a 1% agarose gel and purified using a Monarch DNA Gel Extraction Kit (T1020S, New England Biolabs). The Gibson reaction mixture was transformed into *E. coli* Top10 competent cells to produce high copy number of the fusion plasmid. The expression plasmid was subsequently purified from *E. coli* Top10 and transformed into *E. coli* JIR702 to induce the multimeric form of the plasmid. The presence of the sactipeptide gene cluster in the fusion plasmid was confirmed by PCR amplification using a second pair of primers, 23sac-test_F (5′-ATAATTACCAATTCCCGTTGC-3′) and 23sac-test_R, (5′-GTAAGGAATTAGTATGGGTGAAC-3′) and by restriction enzyme mapping.

### 2.5. Transformation of the SacP23 expression plasmid into *Bacillus subtilis* BS34A by natural transformation

*Bacillus subtilis* is more efficiently transformed by multimeric plasmids compared to monomeric forms [31]. The multimeric fusion plasmid DNA from *E. coli* JIR702 was transformed into *B. subtilis* BS34A via natural transformation. Competent *B. subtilis* BS34A cells were prepared using a modified version of the method described by Harwood & Cutting [32]. Briefly, freshly grown colonies from a TSA plate were suspended in 10 ml of half strength SpC media (0.1 ml l^−1^ 50% glucose; 0.1 ml l^−1^ 2% MgSO_4_; 0.25 ml l^−1^ 1% casamino acids; 0.2 ml l^−1^ 10% nutrient broth; 0.05 ml l^−1^ 1% amino acid solution) in a 50 ml Falcon tube to a final OD_600_ of approximately 0.2. The suspension was incubated at 37°C at 275 rpm until it reached early stationary phase, then 2 ml of the culture was diluted in 10 ml of SpT medium (0.1 ml l^−1^ 50% glucose; 0.41 ml l^−1^ 2% MgSO_4_; 0.1 ml l^−1^ 1% casamino acids; 0.1 ml l^−1^ 10% nutrient broth; 0.05 ml l^−1^ 1% amino acid solution) in a fresh flask and incubating at 37°C for 80 min at 275 rpm.

For transformation, 1–3 µg of plasmid DNA was add to 1 ml of competent cells. The tubes were then shaken at 37°C for 30 min at 200 rpm. The cells were harvested by a low-speed centrifugation at 4500*g* for 10 min, resuspend in 1.5 ml of LB broth and incubated with shaking at 37°C for 90 min, and plated on TSA plates supplemented with 5 µg ml^−1^ chloramphenicol. The presence of the expression plasmid in transformants was confirmed by PCR of the SacP23 structural gene within the bacteriocin gene cluster, and by sequencing of the PCR product.

### 2.6. Enhancement of SacP23 expression by refinement of media and growth conditions

The *B. subtilis* transformants were grown in two types of culture media; production broth (PB), and LB broth; to observe the production of the antimicrobial compound. The medium composition of PB and its use for bacteriocin production from *Bacillus* species was described previously [33]. All cultures and solid media were supplemented with 5 µg ml^−1^ chloramphenicol to maintain the expression plasmid. An MRSA strain, which was also resistant to chloramphenicol, was used as an indicator in antimicrobial detection assays.

Growth curves of bacterial isolates were investigated to quantity antimicrobial activity. To determine the kinetics of bacterial growth, a starter culture was first prepared by inoculating a single colony into 10 ml of selected broth, incubated at 30°C and 200 rpm overnight. One ml of this starter culture was inoculated into 100 ml of fresh media, grown at 30°C and 150 rpm. Five ml culture samples were removed every 6 h, up to 36 h, optical density (OD)_600_ and pH measurements were performed, and then cultures were centrifuged at 4500*g* for 20 min. The supernatant was filtered through a 0.45 µm polyethersulfone membrane to obtain cell-free supernatant. These cell-free supernatants were used in well-diffusion and spot-on-lawn assays to detect antimicrobial activity to determine the point in the growth curve at which the bacteriocin production was at a maximum.

### 2.7. Detection of SacP23 sactipeptide activity by well diffusion and spot-on-lawn assays

Antimicrobial activity in the cell-free culture supernatant, prepared from recombinant *B. subtilis* BS34A(pDLL202Δ-sacP23) cells, was evaluated by well-diffusion assay against MRSA [34]. In brief, Muller Hilton agar (MHA) plates were spread with 0.9% saline containing 1.0 × 10^7^ cfu ml^−1^ of the MRSA strain using sterile cotton wool. Wells were made in the agar using a 6 mm cork-borer. Then, 50 µl of culture supernatant prepared from recombinant *B. subtilis* BS34A(pDLL202Δ-sacP23) cells were applied to the wells and left to completely absorb into the agar, then incubated at 30°C for 12–18 h. Inhibitory activity appeared as a clear zone around the well.

The activity of sactipeptide from purification fractions was evaluated by a spot-on-lawn method. In brief, MHA with half strength agar (0.75%), held at approximately 50°C, was inoculated with MRSA at a cell-density of approximately 1.0 × 10^7^ cfu ml^−1^, mixed well, and then poured into plates. Once the plates had set and been dried, 5–10 µl of each purification fraction was applied to the agar surface, left until completely absorbed, and then incubated at 30°C for 12–18 h. Inhibitory activity appeared as a clear zone around the sample spot.

### 2.8. Purification of the SacP23 sactipeptide by ammonium sulfate precipitation and reverse phase high performance liquid chromatography

The purification procedure included two steps; precipitation of sactipeptide from the culture supernatant with a 70% saturated concentration of ammonium sulfate followed by two cycles of high performance liquid chromatography (HPLC) using a reverse-phase (RP) column (C18). In brief, ammonium sulfate was added to 800 ml cell-free LB supernatant to a final 70% saturation concentration and kept at 5°C overnight. The mixture was centrifuged at 17 500*g* for 90 min at 4°C, and the protein pellet was collected and dissolved in 4 ml of milli-Q water, then desalted and concentrated to a final volume of 500 µl using a centrifugal filter (Millipore) with a protein size cut off of 2 kDa. Next, the material was purified by two cycles of C18 RP-HPLC. After the first RP-HPLC run the fraction with the most antimicrobial activity was concentrated by SpeedVac (at 37°C) and loaded into the RP-HPLC as input for the second cycle of RP-HPLC purification. During purification, MRSA was used as an indicator, using the spot-on-lawn method, to detect the fractions that exhibited antimicrobial activity. The HPLC-purified peptide was submitted for automated Edman degradation analysis for determination of amino-terminal sequence.

### 2.9. Analysis of mature SacP23 sactipeptide by mass spectrometry

Matrix-assisted laser desorption/ionization time-of-flight mass spectrometry (MALDI-TOF-MS) was performed to determine the molecular weight (MW) of the purified sactipeptide using an Autoflex Speed MALDI-TOF instrument (Bruker, Germany). Matrix solution (10 mg of α-cyano-4-hydroxycinnamic acid dissolved in 70% acetonitrile containing 0.1% (v/v) trifluoroacetic acid), was mixed with an equal volume of the purified sactipeptide and 1 µl of the mixture was spotted onto the target plate, air dried and analysed. The molecular ion of mature sactipeptide was detected in the positive ion mode.

### 2.10. Sacp23 sactipeptide characterization by enzymatic stability, thermal stability and antimicrobial activity spectrum

To test the thermal and enzymatic stability of the recombinant bacteriocin, aliquots of the purified sactipeptide were heated at 40°C, 50°C, 60°C, 70°C, 80°C, 90°C and 100°C for 1 h, or incubated with pronase E, proteinase K, trypsin, lipase and catalase, at final concentrations of 1 mg ml^−1^, for 1 h. All enzymes were from Sigma Aldrich. The remaining activity of the sactipeptide, after treatments, was estimated using the spot-on-lawn method, along with untreated samples as controls. To determine the antimicrobial activity spectrum, sactipeptide was tested against a range of indicator strains, including *Lactobacillus plantarum* A6, *Bacillus cereus* ATCC10876, *S. aureus* ATCC25923, MRSA 344/2-24, VRE 345/19, *Listeria monocytogenes* 192/1-2 ACM 3173, *Clostridium perfringens* 52/6-1, *Campylobacter jejuni* ATCC 81116, *Salmonella* Enteritidis ATCC 13076, *E. coli* ATCC25922, and *B. subtilis* BS34A. Strains were obtained from American Type Culture Collection (ATCC) and RMIT University Culture Collection (Australia).

## 3. Results

### 3.1. Analysis of the whole genome sequence of *Paenibacillus polymyxa* #23

The draft genome of *P. polymyxa* #23 has a size of approximately 6.01Mb with a G + C content of 45.2%. The genome sequence has been deposited at DDBJ/ENA/GenBank under the accession JARJLH000000000. The RAST annotation identified 5236 predicted coding sequences. Fifteen putative antimicrobial producing BGCs were found, including 10 non-ribosomally synthesized peptides (NRPs) and five RiPPs—bacteriocins (table 2).

**Table 2.** Predicted antimicrobial compound production encoded by the *P. polymyxa* #23 genome. (The bold letters in bacteriocin sequence indicate the position of the predicted mature/core sequence and the normal font letters denote the leader sequence. Signal sequence predictions were made using SignalP 5.0 (http://www.cbs.dtu.dk/services/SignalP-5.0/). The molecular weight (MW) of the mature/core sequence was calculated using the expasy tool (https://web.expasy.org/compute_pi/).)

	types	most similar known gene cluster (% sequence identity)	precursor bacteriocin sequences/polymer prediction	MW (kDa)	most known peptide (% identity)
**bacteriocin**					
1	Bac1 (lassopeptide)	paenodine (100%)	MSKKEWQEPTIEVLDINQTMA**GKGWKQIDWVSDHDADLYNPS**	2.41	paeninodin (100%)
2	Bac2 (lantipeptide)	paenicidine A (100%)	MAENLFDLDIQVNKSQGSVEPQ**VLSIVACSSGCGSGKTAASCVETCGNRCFTNVGSLC**	3.38	paenicidin A (100%)
3	Bac3 (lantipeptide)	unknown	LPIEVLSTLSKGGIKMNSPELVFFEQEDTLDLDLQINDLTLKQA**KNPCTSTVTCSVSRCLGTHVTCECWC**	2.82	unique
4	Bac4 (sactipeptide)	unknown	MRKLVKRSTNVGDTIEAFG**CGCSCYCPCSCYCAGSLTRSSNTSRESDGSYRRDNGTGIGN**	4.46	unique
5	Bac5 (proteusin)	unknown	unknown	—	unique
**non-ribosomally synthesized antimicrobial peptides**					
1	NRP1	fusaricidine B (100%)	Thr - D-Val - X - D-Thr - D-Asn – X	0.89	fusaricidine B
2	NRP2	polymyxin (100%)	(D-Leu - Leu - Dab - Dab) + (Thr) + (Dab - Thr - Dab)	1.30	polymyxin
3	NRP3	anabaenopetin NZ857/ nostamide A (100%)	—	0.84	anabaenopetin NZ857/ nostamide A
4	NRP4	tridecaptin (80%)	(D-Val - D-Dab)	1.55	tridecaptin
5	NRP5	paenibacterin (60%)	(Trp - Glu) + (Val - D-Ile - X)	1.60	paenibacterin
6	NRP6	tyrocidine (18%) bacillomycin D (20%)	—	—	—
7	hybrid 1 (NRPS-like)	unknown	—	—	—
8	PKS1	nosperin (46%)	—	—	—
9	PKS2	bacillaene (21%)	—	—	—
10	hydrid 3 (transAT-PKS-NRPS)	unknown	—	—	—

Among the NRP, seven of the BGCs were predicted to produce lipopeptides and non-ribosomal peptide synthetase-polyketide synthetase (NRPS–PKS) hybrids and three BGCs encoded polyketide synthases (PKS). Among the lipopeptide BGCs, five BGCs (NRP1 to NRP5) were found to represent known biosynthetic pathways, based on comparisons with the MIBiG database. Whole BGCs of NRP1, NRP2 and NRP3 had 100% gene content similarity with the fusaricidin B BGC (BGC0001152, *P. polymyxa*) [35], polymyxin B BGC (BGC0000408, *P. polymyxa*) [36], and the anabaenopeptin NZ857/nostamide A BGC (BGC0001479, *Nostoc punctiforme* PCC 73102) [37]. The NRP4 BGC had 80% gene similarity with the tridecaptin BGC (BGC0000449, *Paenibacillus terrae*) [38]. The NRPS component, encoded by the core biosynthetic genes, shared 88% amino acid similarity to *TriD* tridecaptin NRPS (AHF21228.1, *P. terrae*). Of the other NRPs, four had differing but lower levels of homology to previously identified clusters and two had no obvious homology to any other BCGs in the databases. The NRP5 BGC had 60% gene similarity with the paenibacterin gene cluster (BGC0000400*, Paenibacillus* sp. OSY-SE) [39]. However, two NRPS core sequences within the gene cluster had 90.0% and 89.0% of amino acid similarity with *TriD* (AHF21228.1) and *TriE* (AHF21229.1) within the tridecaptin BGC (BGC0000449), rather than the paenibacterin synthetase A (AGM16412.1, 52% of similarity) and paenibacterin_synthetase_B (AGM16413.1, 50% of similarity) from a known paenibacterin BGC (BGC0000400). The NRP6 BGC was less similar to known BGCs; it had only 18% similarity with the tyrocidine BGC (BGC0000452, *Brevibacillus brevis* NBRC 100599). Its NRPS had 49.0% similarity with *PlpE* (AFJ14794.1), and 47% similarity to tyrocidine_synthetase_III (BAH43766.1). It indicated that the NRP6 BGC was likely to synthesize a previously unknown lipopeptide. Its NRPS shared 31% sequence similarity with *BogE* (ATY37592.1), a NRPS from the bogorol A BGC (BGC0001532, *Brevibacillus laterosporus*).

**Figure 1.**
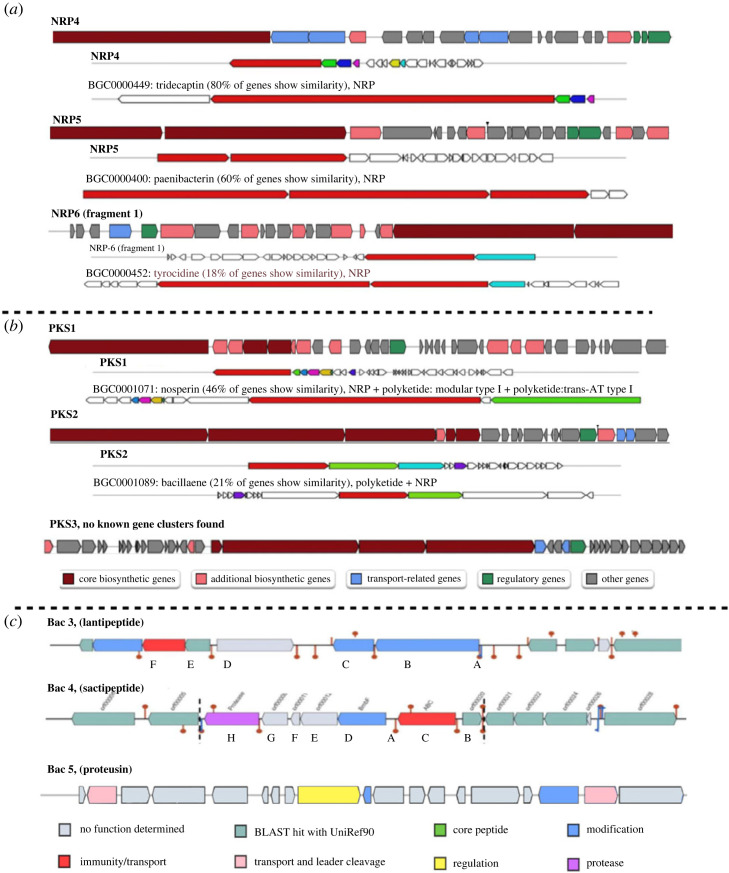
Structural organization of uncharacterized non-ribosomal peptides (*a*), polyketides (*b*) and bacteriocins (*c*) predicted from the *P. polymyxa* #23 genome (the top, thicker line for each gene cluster), in comparison with the most similar known gene clusters from the MIBiG database as defined and presented by antiSMASH software (the thinner lines labelled with percentage of genes showing similarity to the indicated gene cluster). The colour code for (*a*) and (*b*) is at the bottom of (*b*). In (*c*), bacteriocins 3 and 4 show the gene clusters as predicted and rendered by BAGEL software. Bacteriocin 5 was not found by BAGEL and so the gene organization for the predicted proteusin-like encoding gene cluster, as predicted by antiSMASH software, is shown. Bacteriocin encoding gene clusters in which the structural gene is 100% identical to previously characterized bacteriocins are not shown (bacteriocins 1 and 2).

The PKS BGCs had low levels of similarity to known BGCs and so are probably involved in biosynthesis of yet to be characterized peptides. Many polyketides are clinically important agents, with antimicrobial, anticancer and immunosuppressive activity [40,41]. The identification of conserved ketosynthase (KS) domains within these PKS core proteins suggested an association with PK production [42]. Among the PKS BGCs (figure 1*b*), the PKS1 BGC had 46% of gene cluster similarity to the nosperin BGC (BGC0001071, *Nostoc* sp*.*) [43] but the structural organization of module components was different. Three predicted PKS within this gene cluster shared highest sequence similarity with a hybrid NRPS/PKS protein (CAG23957.2, 41% of similarity), 3-hydroxy-3-methylglutaryl CoA_synthase (CAG23954.2, 78% of similarity), and calW (BAP05575.1, 63% of similarity). The PKS2 BGC may be fragmented into two short contigs containing fragments of transAT-PKS synthetases. The whole gene region of the first BGC fragment shared 21% gene similarity to the bacillaene BGC (BGC0001089, *B. velezensis* FZB42) [44] and 7% gene similarity to the aurantinin BGC (BGC0001520, *B. subtilis*) [45]. Four biosynthetic core proteins in the fragment 1 shared highest similarity to a polyketide_synthase_modules-related_protein (AJQ95708.1, 32.0% of similarity), malonyl_CoA-acyl_carrier_protein_transacylase (ATX68109.1, 93%), malonyl_CoA-acyl_carrier_protein_transacylase (AKQ22669.1, 42%), and acyltransferase_acyltransferase_2-nitropropane_dioxygenase_(NPD)-like_domain (RAT98531.1, 44%). The second PKS2 BGC fragment shared 14% gene similarity to the pyxipyrrolone A BGC (BGC0001751, *Pyxidicoccus* sp.) [46] and 10% to aurantinin BGC (BGC0001520, *B. subtilis*) [45]. Two PKSs in the second fragment shared 53% sequence similarity (ERM18797.1), and 57% similarity (ERM18797.1) with a known PKS, respectively. The PKS3 BGC was found to be related to an uncharacterized polyketide. Nine core biosynthetic proteins within the gene cluster shared low sequence similarity (less than 53%) to PKS. The NaPDoS-based analysis of these KS domains of the core biosynthetic enzymes found the sequence similarity of these domains with the sequences in the database was ≤56.0%. However, a similarity of 100% of these KS domains at the amino acid level (based on BlastP search against non-redundant database) is necessary for prediction as the putative product of PKS [47]. Within the draft genome sequence other NRPS gene fragments were noted in a range of short contigs. These fragmented BGCs remain to be fully characterized following future genome completion and closure.

The five bacteriocin BGCs predicted from the *P. polymyxa* #23's genome sequence are all class I bacteriocins, including two lanthipeptides, one lassopeptide, one sactipeptide, and one bacteriocin of the proteusin family. The Bac1 (lantibiotic) and Bac2 (lassopeptide) BGCs had 100% of the genes previously characterized in the BCGs encoding paenicidin A synthesis and processing (BGC0000541), and the paeninodin encoding BGC (BGC0001356, *Paenibacillus dendritiformis* C454), respectively. The remaining bacteriocin BGCs were found to be associated with biosynthesis of unknown bacteriocins. Their precursor amino acid sequences were unmatched to any characterized bacteriocins from the bacteriocin database (BACTIBASE) [29] and uniref 90 database (UNIPROT). All bacteriocin precursor sequences are shown in table 3 and the structural organization of the gene clusters is illustrated in figure 1*c*. The novel lantibiotic BGC (Bac3) has two genes that encode typical lantibiotic modification enzymes; lanB and lanC [48], thus indicating an association to biosynthesis of a lantibiotic bacteriocin. The lantipeptide precursor is 53 amino acids (aa), consisting of a predicted 29 aa signal sequence at the N-terminus and a 24 aa core sequence at the C-terminus, with a theoretical MW of 2375.2 Da, and unique within the BACTIBASE database. A novel sactipeptide (Bac4) BGC was predicted based on the presence of a radical *S*-adenosyl-l-methionine (*S*AM) protein—a typical modification enzyme for sactipeptide bacteriocins. The sactipeptide precursor was 100% identical to an uncharacterized putative bacteriocin CLI_3235 family (A0A378XQ17, *P. polymyxa*), unique on the BACTIBASE database. The presence of a putative nitrile hydratase-like precursor gene within the Bac5 BGC, suggests an association to biosynthesis of a proteusin family bacteriocin—a recently identified family of RiPP natural products. The gene cluster was predicted by antiSMASH software but not by BAGEL 4. Although no structural gene was found within the Bac5 gene cluster, many other necessary components for bacteriocin production were found, including regulation genes, transport-related genes, and additional biosynthetic genes. Members of the proteusin family are mostly identified from *in silico* mining, and few of this class of bacteriocins have been biologically characterized. The first proteusin family representative, polytheonamide, was characterized in 2015, and its biosynthesis pathway was found to result in nearly 50 post-translational modifications [49–51].

**Table 3.** Annotation of the open reading frames in the sactipeptide encoding gene cluster found in *P. polymyxa* #23**.**

proteins	TMH^a^	description	function	similar proteins in uniref90 database
23sacA		precursor bacteriocin	precursor	—
23sacB		ECF subfamily RNA polymerase sigma-24 subunit	unknown	A0A378XN73, (99.3%); *P. polymyxa*
23sacC	6	multidrug ABC transporter protein	transportation	A0A378XMM5 (100%); *P. polymyxa*
23sacD	0	cys-rich peptide radical *S*AM maturase TIGR04068	modification of precursor	A0A2S6NY22 (95.4%); *P. peoriae*
23sacE	0	protein with domain TIGR04066	immunity	UPI000D2F5CC2 (10%); *P. polymyxa*
23sacF	0	peptide maturation system acyl carrier-related protein (TIGR04069)	modification of precursor	A0A378XN50 (100%); *P. polymyxa*
23sacG	0	muscle M-line assembly protein unc-89 uncoordinated protein 89	unknown	A0A378XQ26 (100%); *P. polymyxa*
23sacH	0	subtilin-like serine protease	removal of leader sequence	A0A378XQL5 (100%); *P. polymyxa*

^a^TMH, number of transmembrane helices predicted by the TMHMM server, v.2.0 (http://www.cbs.dtu.dk/services/TMHMM/).

### 3.2. Characterization of the novel sactipeptide biosynthetic gene cluster encoding SacP23

**Figure 2.**
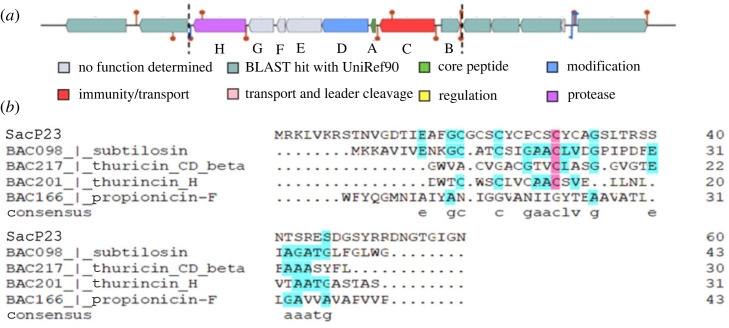
(*a*) The gene cluster encoding sactipeptide synthesis and processing. (*b*) Multiple sequence alignment between the SacP23 sactipeptide precursor and known sactipeptide precursors. The multiple sequence alignment was calculated by DNAman software using Clustal W. The letters are in black (100% of identity), pink (≥70%) and blue (50%). Sactipeptides were downloaded from the BACTIBASE database.

BCG 4 spanned 8556 bp and consisted of eight open reading frames encoding eight proteins, SacP23ABCDEFGH (figure 2). The presence of a gene (23*sac*D) encoding a radical *S*AM enzyme within the gene cluster indicated that the cluster related to production of a sactipeptide [52]. Figure 2*b* shows the sequence of the structural gene encoding the precursor bacteriocin (SacP23). It consisted of 61 aa comprising a 19 aa leader sequence and a 42 aa mature peptide. The precursor sactipeptide is 100% identical to an uncharacterized putative bacteriocin, CLI_3235 family (A0A378XQ17-*P. polymyxa*) but unique on the BACTIBASE database. The 42 aa core sequence is cysteine-rich with a theoretical MW of 4455 Da and a pI of 7.73 by prediction with SignalP 5.0. Other accessory genes predicted to be involved in SacP23 production and processing are listed in table 3. By analogy to other bacteriocin production loci, the maturation of the sactipeptide is probably initiated by a subtilase-family serine protease (encoded by 23*sac*H) responsible for cleavage of the signal sequence [53], followed by modification of the core sequence by two enzymes; the radical *S*AM protein (encoded by 23*sac*D) and an acyl carrier protein homologue (TIGR04069) (encoded by 23*sac*F). The matured peptide may then be secreted by a putative ABC transporter, 23*sac*C. The 23*sac*E gene encodes a hypothetical protein belonging to the TIGR04066 family. This protein family contains subunits of an H + -transporting two-sector ATPase, suggesting a function in immunity to 23SacE. The 23*sac*G gene was annotated as ‘muscle M-line assembly protein unc-89 uncoordinated protein’, with unknown function in bacteriocin biosynthesis.

### 3.3. Cloning of the SacP23 sactipeptide gene cluster and expression in *Bacillus subtilis*

**Figure 3.**
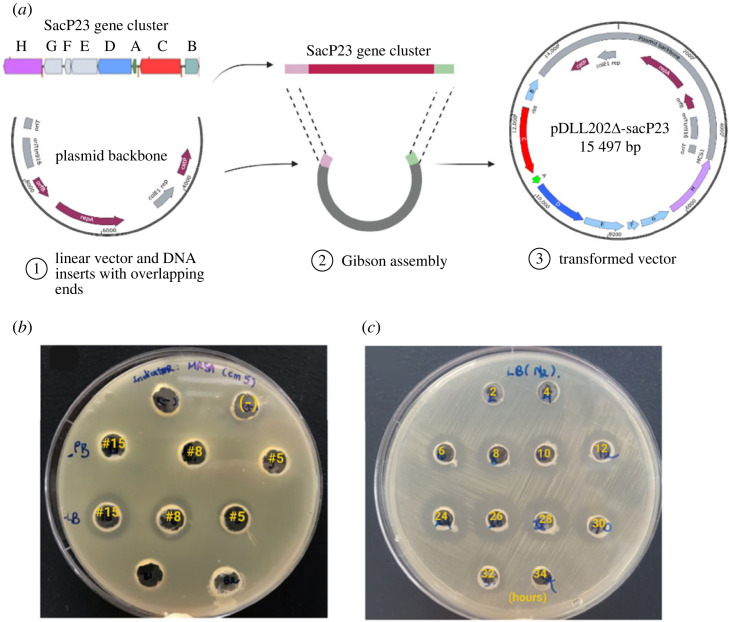
(*a*) Cloning of the SacP23 gene cluster into the pDLL202 shuttle vector to produce the heterologous expression plasmid pDLL202Δ-sacP23. (*b*) Cultivation of three *B. subtilis* BS34A(pDLL202Δ-sacP23) transformants (nos. 5, 8, 15) in PB (second row) and LB broth (third row). The antimicrobial activity (zone of clearing) was only seen in cultures grown in LB media. A culture of the *B. subtilis* harbouring the plasmid vector, pDLL202, was used as a negative control. (*c*) Antimicrobial activity exhibited by *B. subtilis* BS34A(pDLL202Δ-sacP23) LB grown culture—supernatant collected at the different times shown.

The 8556 bp DNA fragment containing the whole sactipeptide gene cluster, including the upstream region expected to carry the promoter region for the BCG, was inserted into the 6938 bp Spe1/MluI digested backbone of the shuttle vector plasmid, pDLL202 (figure 3*a*). The resulting plasmid, pDLL202Δ-sacP23, with a size of 15 497 bp, was transformed into *E. coli* Top10 and then moved to *E. coli* JIR702 to produce the multimeric form of the plasmid. Multimeric plasmid was then used to transform *B. subtilis* BS34A. The accuracy of construction was tested by restriction enzyme analysis and PCR amplification of regions within the sactipeptide gene cluster.

Expression of antimicrobial activity by the *B. subtilis* BS34A(pDLL202Δ-sacP23) transformants was assessed in two types of culture media: PB; and LB broth. The production of sactipeptide was only seen in LB broth (figure 3*b*). Antimicrobial activity was first detected after 6 h of culture growth and the peak in activity occurred at 24 h of incubation, corresponding to a culture density of approximately 1.4 (OD_600_), after which the activity reduced and was lost after 32 h of incubation. Therefore, a 24 h culture was prepared from which to purify and characterize the antimicrobial compound.

### 3.4. Two-step purification of the SacP23 sactipeptide

**Figure 4.**
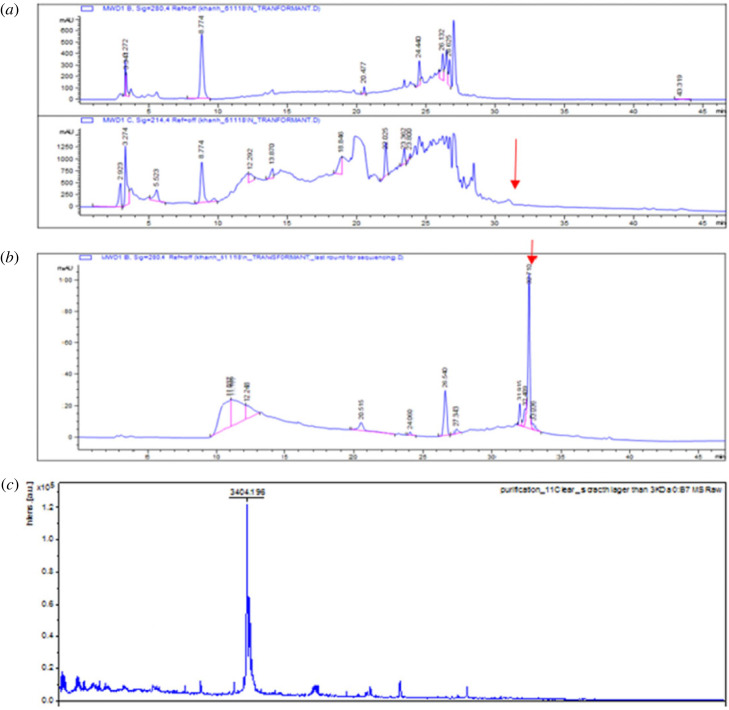
RP-HPLC purification of the ammonium sulfate precipitate of culture supernatant. (*a*) The first round of RP-HPLC resulted in antimicrobial elution at t*R* of 30–32 min. (*b*) The second round of RP-HPLC resulted in antimicrobial elution at t*R* of 32.77 min. (*c*) MALDI-TOF-MS of purified SacP23 sactipeptide. The major peak of SacP23 was at *m/z* 3404.

The purification procedure included two steps: precipitation of SacP23 sactipeptide from the culture supernatant with 70% saturated ammonium sulfate, followed by purification through two cycles of C18 RP-HPLC. The first RP-HPLC cycle, a 50 min gradient elution that started at 2% acetonitrile + 98% H_2_0 and ended with 100% acetonitrile, resulted in an antimicrobial fraction at a retention time of 30–32 min (figure 4*a*). The second round of RP-HPLC used a 60 to 70% acetonitrile gradient and resulted in the elution of an antimicrobial compound at a retention time of 32.7 min (figure 4*b*). The MALDI-TOF-MS revealed a major peak with *m/z* 3404 Da (figure 4*c*). Attempts to obtain amino-terminal sequence data using automated Edman degradation were unsuccessful, indicating a blocked N-terminus.

### 3.5. Properties of the recombinant SacP23 sactipeptide

**Figure 5.**
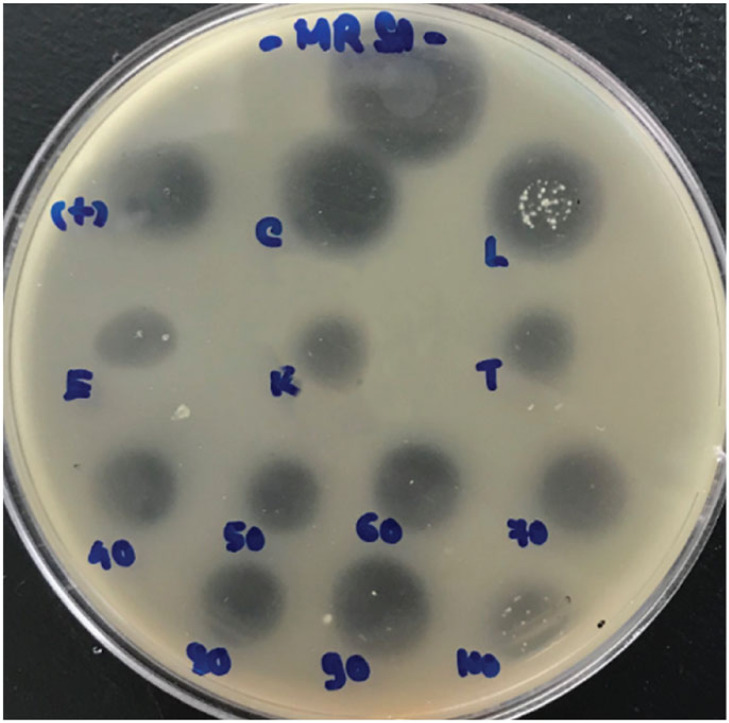
Thermal and enzymatic stability of recombinant SacP23. Enzymatic sensitivity to: catalase (C), lipase (L), pronase E (E), proteinase K (K) and trypsin (T) was evaluated by comparing to undigested SacP23 (+). The thermal stability was determined by heating at 40°C, 50°C, 60°C, 70°C, 80°C, 90°C and 100°C for 1 h. Only the protease treatments and heating at 100°C noticeably reduced SacP23 activity.

The recombinant SacP23 was resistant to high temperature treatment, with activity maintained after treatment at 100°C for 1 h, although the activity was somewhat reduced. The peptide was sensitive to pronase E, proteinase K, and trypsin, but was resistant to lipase and catalase (figure 5). The buffer in which SacP23 was resuspended, and the proteases, lipase, and catalase, did not produce any zones of activity against the indicator bacteria (result not shown). The recombinant SacP23 sactipeptide exhibited antimicrobial activity against several Gram-positive bacteria including an MRSA isolate (table 4). The base *B. subtilis* strain in which expression was achieved was also sensitive to the recombinant SacP23 sactipeptide. This indicates that the predicted 23SacE immunity protein was also expressed and effective in the recombinant strain, allowing it to survive in the face of sactipeptide expression. No activity was seen against Gram-negative bacteria (table 4).

**Table 4.** Antimicrobial spectrum of purified SacP23 sactipeptide.

indicators	media used for test	antimicrobial activity^a^
*S. aureus* ATCC 25923^b^	MHA	+
*S. aureus* MRSA 344/2-24^c^	MHA	+
*B. subtilis* BS34A^c^	MHA	+
*B. cereus* ATCC 10876^b^	MHA	+
*L. monocytogenes*192/1-2 ACM 3173^c^	MHA	−
*C. perfringens* EHE-NE18	MHA	−
*L. plantarum* A6^c^	deMan, Rogosa and Sharpe (MRS)	−
*E. faecalis* (VRE) 345/19^c^	MRS	+
*S.* Enteritidis ATCC 13076^b^	MHA	−
*E. coli* ATCC 25922^b^	MHA	−
*C. jejuni* ATCC 81116^b^	MHA + 5% sheep blood	−

^a^Positive inhibitory activity [+], no inhibitory activity [−].

^b^Strains obtained from American Type Culture Collection (ATCC).

^c^Strains obtained from RMIT University Culture Collection.

## 4. Discussion

The rapid spread of antibiotic-resistant pathogens in clinical and agricultural settings has led to an urgent need for alternative novel antimicrobials. Bacteria are a rich source of antimicrobial compounds and analysis of isolates from understudied sources, such as the marine environment, have identified bacterial isolates that encode the synthesis of extensive repertoires of antimicrobial compounds [22,54,55]. The marine derived isolate *P. polymyxa* #23 was previously identified as expressing broad antimicrobial activity against a range of both Gram-negative and Gram-positive bacteria [22]. This study has now identified the genetic basis for this broad antimicrobial activity. From an analysis of the whole genome sequence of *P. polymyxa* #23, it was found that a diverse collection of bacteriocins and secondary metabolite antimicrobial compounds is encoded by the strain. Interestingly, many of the predicted antimicrobials appeared to be uncharacterized, and hence they represent novel antimicrobial peptides. The strong and broad antimicrobial activity exhibited by *P. polymyxa* #23 is likely to be a consequence of additive and possibly synergistic effects of the various antimicrobial compounds. The homologues of previously identified antimicrobial compounds could account for much of the antimicrobial activity exhibited by the strain. For example, the fusaricidin B lipopeptide has been shown to have inhibitory activity against many phytopathogens and Gram-positive bacteria [56]. Tridecaptin has strong activity against many Gram-negative bacteria including colistin-resistant *Klebsiella pneumonia*, *E. coli,* and *Campylobacter* species [57]. Polymyxin can suppress the growth of *Pseudomonas aeruginosa* and *K. pneumonia* [58]. Currently, these two antimicrobial compounds are widely used in the clinic for treatment of serious Gram-negative bacterial infections. The paenibacterin lipopeptide is a broad spectrum bactericidal agent with antimicrobial activity against both Gram-negative and Gram-positive pathogens including antibiotic-resistant strains of *E. coli*, *P. aeruginosa, Acinetobacter baumannii, K. pneumoniae, S. aureus* and *Enterococcus faecalis* [59]. Other activity against Gram-positive bacteria may be derived from the bacteriocins paenicidine A and paeninodin and the potentially novel bacteriocins identified in this study. Although not tested in this study, *P. polymyxa* #23 may exhibit antiviral activity owing to the presence of a predicted proteusin family member and polyketides. Proteusin bacteriocins have mostly remained as *in silico* predictions, with only a few isolated and characterized. A proteusin family representative, polytheonamide, was recently characterized from sponge-associated bacteria [50]. Another member of proteusin family, landornamide A, was isolated from a *Cyanobacteria* sp. with antiviral activity [60].

With the development of next generation DNA sequencing methods and the subsequent generation of large databases of bacterial genome sequences, there is now an extensive collection of genes that are predicted to encode the synthesis of antimicrobial compounds. Most of these have remained as predictions without any biological validation of expression or antimicrobial activity. If this treasure trove of novel antimicrobial compounds is to be exploited, then more physical and biological analysis needs to be undertaken to define the antimicrobial activity spectrum of novel compounds. To assess the individual antimicrobial compounds from organisms such as *P. polymyxa* #23, that encode multiple relevant compounds, they need to be separated and purified. This can be done biochemically or genetically. Biochemical separation and analysis can sometimes be difficult if strains express several different compounds with similar physical properties. Also, some predicted antimicrobial compounds are not produced under standard laboratory culture conditions and hence are not readily available for purification and analysis. In this study the novel sactipeptide, SacP23, was characterized following expression and purification from a heterologous expression system. Cloning of the complete SacP23 gene cluster enabled the separation of SacP23 expression away from all the other antimicrobial compounds produced by *P. polymyxa* #23.

Purified SacP23 was found to have high heat stability and was not adversely affected by lipase or catalase but was inactivated by protease treatment, as would be expected for a peptide antimicrobial. Bioinformatic analysis had predicted that the mature SacP23 peptide should have an MW of 4.46 kDa, yet the MALDI-TOF MS analysis of the purified recombinant SacP23 showed an MW of 3.4 kDa. This indicates that the peptide is processed further than the simple removal of the predicted amino-terminal leader sequence, either by further trimming at the amino or carboxy termini. A similar finding of unpredicted processing of a bacteriocin has been reported for lactolisterin BU [61]. Attempts to characterize the amino-terminal end of the peptide were unsuccessful, indicating that there was some form of modification that was resistant to Edman degradation analysis. The failure of N-terminal sequencing has been frequently reported for highly modified bacteriocins such as the sactipeptides [23] and the circular bacteriocins, such as enterocin AS-48, subtilosin A, circularin A and closticin 574 [62–64]. The expectation is that, as in other sactipeptides, all or some of the cysteine residues in SacP23 are likely to be involved in the formation of intramolecular thioether linkages, but this remains to be elucidated.

Sequence alignment between SacP23 and previously characterized sactipeptides (obtained from BACTIBASE database) showed that SacP23 is most closely related to subtilosin A (figure 3*b*). Both these two bacteriocins have the same numbers of amino acids residues and have similar MWs of approximately 3–4 kDa. Therefore, this discrepancy in MW may be a result of a misprediction of the SignalP 5.0 software that was used to predict the signal sequence cleavage position on the precursor, subsequently causing misprediction of the MW of the mature sequence. To investigate this further, sequences of well characterized bacteriocin precursors (amylocyclicin, mersacidin and paenicidin A) were submitted to the software for prediction of cleavage sites. The software suggested incorrect recognition sites, leading to an incorrect prediction of the MW of mature sequence. Although SignalP 5.0 is widely used for this purpose, it is clear that the software is sometimes ineffective in the case of small peptides like bacteriocins. Further physical analysis of the bioactive SacP23 peptide will need to be undertaken to fully define the structure of the mature sactipeptide.

The heterologously expressed recombinant SacP23 sactipeptide exhibited antimicrobial activity against some Gram-positive bacteria, importantly including strong activity against an MRSA isolate. Further studies need to be undertaken to fully assess the potency of the SacP23 by performing minimum inhibitory concentration tests for a range of sensitive bacteria. The most closely related sactipeptide, subtilosin A, is reported to have antimicrobial activity extending to both Gram-positive and Gram-negative bacteria [65]. This indicates the diversity in antimicrobial spectra of sactipeptide-type bacteriocins and shows that it may be possible to tailor their use to specific applications—for example treating MRSA infections without adversely affecting the resident commensal bacterial populations.

## 5. Conclusion

The identification of a diverse array of antimicrobial peptides encoded by *P. polymyxa* #23 suggests that this strain, or individual antimicrobial products from it, may have therapeutic potential and could be used in medical, agricultural, and food industries in the future. The study has shown that cloning and heterologous expression is an ideal approach to fully characterize each of the antimicrobial peptides in isolation from all the other peptides predicted to be produced by bacteria such as this, that encode the synthesis of many antimicrobial products. The purified novel sactipeptide, SacP23, can now be further characterized, both in terms of structural analysis and antimicrobial activity spectrum.

## Data Availability

*Paenibacillus polymyxa* #23 whole genome DNA sequences: Genbank accession JARJLH000000000 (https://www.ncbi.nlm.nih.gov/nuccore/JARJLH000000000.1/).
